# From Data towards Knowledge: Revealing the Architecture of Signaling Systems by Unifying Knowledge Mining and Data Mining of Systematic Perturbation Data

**DOI:** 10.1371/journal.pone.0061134

**Published:** 2013-04-23

**Authors:** Songjian Lu, Bo Jin, L. Ashley Cowart, Xinghua Lu

**Affiliations:** 1 Department of Biomedical Informatics, University of Pittsburth, Pittsburgh, Pennsylvania, United States of America; 2 Department of Biochemistry and Molecular Biology, Medical University of South Carolina, Charleston, South Carolina, United States of America; University of Catania, Italy

## Abstract

Genetic and pharmacological perturbation experiments, such as deleting a gene and monitoring gene expression responses, are powerful tools for studying cellular signal transduction pathways. However, it remains a challenge to automatically derive knowledge of a cellular signaling system at a conceptual level from systematic perturbation-response data. In this study, we explored a framework that unifies knowledge mining and data mining towards the goal. The framework consists of the following automated processes: 1) applying an ontology-driven knowledge mining approach to identify functional modules among the genes responding to a perturbation in order to reveal potential signals affected by the perturbation; 2) applying a graph-based data mining approach to search for perturbations that affect a common signal; and 3) revealing the architecture of a signaling system by organizing signaling units into a hierarchy based on their relationships. Applying this framework to a compendium of yeast perturbation-response data, we have successfully recovered many well-known signal transduction pathways; in addition, our analysis has led to many new hypotheses regarding the yeast signal transduction system; finally, our analysis automatically organized perturbed genes as a graph reflecting the architecture of the yeast signaling system. Importantly, this framework transformed molecular findings from a gene level to a conceptual level, which can be readily translated into computable knowledge in the form of rules regarding the yeast signaling system, such as “*if genes involved in the MAPK signaling are perturbed, genes involved in pheromone responses will be differentially expressed.*”

## Introduction

Model organisms, such as *Saccharomyces cerevisiae* and *Drosophila melanogaster*, are powerful systems for studying cellular signal transduction because they are amenable to systematic genetic and pharmacological perturbations, enabling biologists to infer whether a gene is involved in a signal transduction pathway through studying perturbation-response data. The premise for elucidating signal transduction pathways from systematic perturbation experiments is that, if perturbation of a set of genes consistently causes a common cellular response, e.g., a phenotype presented as the differential expression of a module of genes, the perturbed genes are likely the members (or modulators) of the signal transduction pathway that leads to the phenotype.

In this study, we refer to a *signal* from an information theory [1] point of view, in which a signal is a latent variable whose state contains information with respect to another variable, e.g., the expression state of a gene module or the state of another signal. From the same viewpoint, a signaling system consists of a set of latent variables connected as a network, in which an edge exists between a pair of signals if the state of one signal affects that of the other, i.e., information can be transmitted between the signals, and the relay of signals along the paths in the network enables the system to encode complex information. From a cell biology viewpoint, a signal transduction pathway consists of a collection of signaling molecules that detect and transmit a signal that has a physical or chemical form, e.g., the presence of pheromone in the environment. In such a system, a signal is encoded as a change in the state of a signaling molecule, often manifested as a change in the structural conformation of a protein, a chemical modification of a signaling molecule, or a change in the concentration of a signaling molecule. While it would be ideal to find a one-to-one mapping between the signaling molecules in cells and the signals in the information theory framework, such a mapping can be difficult to obtain and too complex to represent. Representing cellular signaling systems within the abstract information-theory framework provides the following advantages: 1) it enables us to use latent variables to represent the state of yet unknown signaling molecules; 2) it allows us to represent the biological signals encoded by a group of signaling molecules into a single-bit signal, if the signals encoded by these molecules convey a common piece of information with respect to other variables. We refer to such a group of signaling molecules as a *signaling unit*. The following example illustrates the parallelism between the biological entities and and their counterparts in a computational model. A pheromone receptor in a yeast cell and its associated G-proteins can be thought of as one signaling unit, as they function together inseparably to detect the signal of a pheromone. Another exemplary signaling unit is the cascade of mitogen-activated protein kinases (MAPKs), which transduce signals among themselves through a chain of protein phosphorylation reactions almost in a deterministic fashion. The states of these signaling units can be represented as two single-bit signals in a computational model. When a yeast cell is exposed to pheromone, the receptor unit detects the signal and transmits it to the MAPK unit [2], [3], which further relays the signal to downstream signaling units to regulate the expression of downstream genes involved in mating. These relationships between signaling units can be represented as edges in the model. Moreover, in addition to pheromone response, the MAPK signaling unit also interacts with other signaling units to transmit the signals that affect filamentation/invasion processes [2], [3]; such branching and cross-talking between different signaling pathways can be represented as a network of connected signals in the computational model. Thus, the general task of using systematic perturbation data to study a cellular signaling system can be reduced to the following specific tasks: 1) revealing the signals embedded in the convoluted molecular phenotype data such as microarrays; 2) identifying perturbed genes that affect a common signal; 3) grouping perturbed genes into signaling units based on the information they encode; and 4) inferring the paths between signaling units where a path may or may not correspond to a signal transduction pathway in conventional cell biology.

In the seminal work by Hughes *et al.* [4], yeast cells were subjected to over 300 types of systematic perturbations (gene deletions and chemical treatments (from here on, we refer to such a treatment experiment as a perturbation instance), and the transcriptional responses to the perturbations were measured using microarrays. This dataset has been widely used to test different computational approaches for investigating the relationship between perturbed genes and responding genes [4]–[9]. For example, using a conventional hierarchical clustering approach, Hughes *et al.* [4] grouped perturbed genes into clusters to elucidate the cellular functions of some genes, based on the fact that perturbing these genes produced gene expression profiles similar to those resulting from perturbing the known members of certain pathways. To relax the requirement of global similarity by hierarchical clustering, other researchers have studied approaches to connect a subset of perturbation instances to a subset of responding genes in order to find context specific information between the perturbation and the responses [8]. Such a task is often cast as a biclustering problem [10]–[12]. More recently, sophisticated graph-based algorithms have been applied to the dataset to study potential signal pathways [5], [7], [9]. The basic idea underlying the studies by Yeger-Lotem *et al.* [9] and Huang *et al.* [5] is to model the information flow from perturbed genes to responding genes through a PPI network by employing graph search algorithms, such as price collecting Steiner tree algorithms.

While the above studies have led to many biological insights regarding the system at a gene level, they have not addressed the task of discovering signaling units and representing the findings at a conceptual level in order to derive computable knowledge, such as the rule: *if a gene involved in a MAPK pathway is deleted, the cellular response to pheromone will be affected*. Transforming experimental data into concepts and further elucidating the relationship among the concepts are critical steps of knowledge acquisition and knowledge representation. The scale of contemporary biotechnologies further necessitates computational approaches to automate such tasks in order to facilitate knowledge discovery by human experts. Yet, the development of such techniques is severely lagging behind the pace of data generation. In this paper, we report a proof of concept framework that unifies knowledge mining and data mining to derive knowledge regarding a signaling system in an automatic manner; we refer to the overall approach as ontology-driven knowledge discovery of signaling pathways (OKDSP). We tested the framework using the yeast perturbation-response data by Hughes *et al.* [4] to illustrate its utility.

## Results and Discussion

A key step of “reverse engineering” signaling pathways using systematic perturbations data is to identify perturbations that convey the same information or, in other words, to first find the “jigsaw puzzle” pieces belonging to a signal transduction pathway. For example, a classic yeast genetic approach is to search for deletion strains that exhibit a common phenotype as a means for identifying genes potentially involved in a signaling pathway carrying information with respect to the phenotype [13]. The advent of genome technologies enables biologists to use genome-scale data, such as gene expression data, as “molecular phenotypes” to study the impact of systematic perturbations [4]. In general, a perturbation treatment, such as deleting a gene, often affects multiple biological processes. For example, deleting a gene involved in ergosterol metabolism will affect the organization of cell membrane, which in turn will affect multiple signaling pathways located in the membrane. As such, the overall cellular response to a perturbation instance, which often manifests as a long list of differentially expressed genes, inevitably reflects a mixture of responses to multiple signals. Thus, we are confronted with two fundamental tasks when studying systematic perturbation data: 1) dissecting signals from the convoluted gene expression responses to a perturbation instance; i.e., finding a module of genes whose expression state reflects the state of a signal transduced along a signaling pathway; and 2) identifying a set of perturbation instances that affects the signal regulating a common expression module.

To address the tasks, we hypothesize that, if a module of genes—whose functions are coherently related—responds to multiple perturbation instances in a coordinated manner, the genes in the module are likely regulated by a common signal, and the perturbation instances affect this signal. Based on this assumption, we can first decompose the overall expression response to a perturbation instance into functional modules, with each module potentially responding to a distinct signal; then we can investigate if a functional module is repeatedly affected in multiple perturbation instances. In this study, we developed an ontology-based knowledge-mining approach to identify functional modules, and we then developed a novel bipartite-graph-based data-mining approach to search for perturbation instances affecting a common signal. Based on the results from the steps above, we further identified signaling units and revealed their organization in a signaling system using a graph-based algorithm.

### Identifying functional modules through knowledge mining

The Gene Ontology (GO) [14] contains a collection of biological concepts (GO terms) describing the molecular biology aspects of genes. The relationship among the concepts are represented in a directed acyclic graph (DAG). An edge reflecting an “is-a” relationship between a pair of GO terms indicates that the concept encoded by the parent term is more general and subsumes the concept of the child term. The GO has been widely used to annotate the function of genes of different model organisms; therefore, it is natural to treat a set of genes annotated with a common GO term as a *functional module*, a widely used approach in bioinformatics analyses [15], [16].

**Figure 1:**
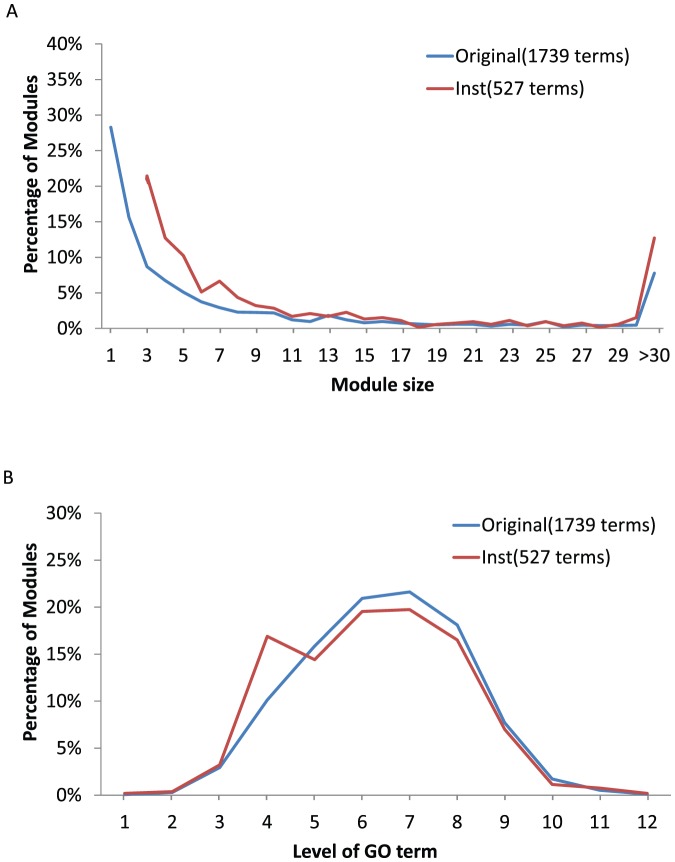
Characterization of the summary GO terms. **A**. The histograms of the number of genes associated with each GO term before and after ontology-guided knowledge mining: 1) the original GO annotations for all responding genes (blue); and 2) the GO terms returned by the instance-based module search (red). **B**. The distribution of the levels of the above GO term sets in the ontology hierarchy are shown as normalized histograms. Level 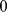 represents the root of the Biological Process namespace.

We first investigated if original GO annotations from the GO database are suitable to represent the major functional themes of genes responding to perturbations in our setting. Based on the results of gene expression analysis performed by Hughes *et al.* [4], 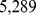 genes were determined to be differentially expressed in response to one or more perturbation instance(s). We identified all the GO terms that have been used to annotate these genes and retained a subset that belong to the Biological Processes domain of the GO, which consists of 1,739 unique GO terms. We studied the distribution of the number of genes annotated by each GO term, and represented the results as a histogram in Figure 1. The figure shows that a large number of original GO annotations were associated with only a few genes; in fact almost half (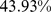) of the GO terms were associated with only 1 or 2 genes. The results reflect the fact that, while original GO annotations are highly specific and informative with regard to individual genes, they would fail to represent the major functional themes of a set of genes. Therefore, there is a need to identify more general terms to represent major functional themes.

We then formulated the task of finding functional modules as follows: given a list of genes responding to a perturbation instance and their GO annotations, assign the genes into non-disjoint functional modules, such that genes within a module participate in coherently related biological processes. This was achieved by utilizing the hierarchical organization of the GO to group a subset of genes under a suitable GO term, one which retains as much of original the semantic information as possible. We developed novel quantitative metrics for objectively assessing the fitness of a summarizing GO term, enabling us to find a term that covered many genes and yet minimized the loss of semantic information from the original annotations [17]. Our criteria for a summarizing GO term included: 1) requiring the summarizing term to be statistically enriched in the input gene list; and 2) requiring the functions of the genes in a module to be semantically coherent when measured with a functional coherence metric previously developed by our group (Richards, *et al.* 2010 [18] and see Methods section). This enabled us to dynamically search for suitable terms along the GO hierarchy and to group genes under suitable summary terms in a manner that is specific for each input gene list, rather than using pre-fixed annotations [16]. We refer to this approach as a knowledge-mining approach because it searches for a new representation of the function of genes through assimilating knowledge represented by the original annotations.

Applying this approach, we identified functionally coherent modules for each perturbation experiment. Further, we merged the modules from different perturbation instances that shared a common GO annotation. The procedure led to a total of 527 distinct functional modules, each summarized with a distinct GO term. The statistics of the modules, the number of genes annotated by summarizing terms, and the levels of the terms in the GO hierarchy, are shown in Figure 1. It is interesting to note that while the summarizing GO terms tend to annotate more genes than the original ones, the distribution of the terms along the GO hierarchy is quite close to that of the original annotations, indicating that our approach retained a level of semantic specificity similar to that of the original annotations.

We further investigated the modules and found the results biologically sensible. For example, we found that 38 genes were grouped into a module annotated with the term GO:0008643 (*carbohydrate transport*) (from here on, we name a functional module using its summary GO term), including 17 genes in the hexose transport {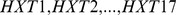}. The original annotations of the genes in the module included GO:0051594 (*detection of glucose*, covering 3 genes), GO:0005536 (*glucose binding*, covering 3 genes), GO:0005338 (*nucleotide-sugar transmembrane transporter activity*, covering 4 genes), GO:0005353 (*fructose transmembrane transporter activity*, covering 16 genes), and so on. Our algorithm summarized the function of the genes using the term GO:0008643 (*carbohydrate transport*), which we believe does not result in a significant loss of information regarding the individual genes, thus providing a sensible representation of the overall function of a larger group of genes. A list of function modules is shown in the supplementary website.

### Searching for perturbation instances affecting a common signal

Using a functional module from the previous section as a putative unit responding to a cellular signal, we further searched for the perturbation instances that affected expression state of the functional module. Success in finding a set of functionally coherent genes that repeatedly co-responded to multiple perturbation instances would provide a strong indication that the responding genes are regulated, as a unit, by a common signal, and that the perturbation instances may have affected such a signal. We addressed the searching task in the following steps: 1) Given a functional module, we first created a bipartite graph placing all perturbation instances on one side and the genes in the functional module on the other side, referred to as a functional-module-based graph. In such a graph, an edge between a perturbation instance and a responding gene indicates that the gene is differentially expressed in response to the instance. 2) We then searched for a densely connected subgraph satisfying the following conditions: a) each vertex was, on average, connected to a given percent, 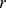, of the vertices on the opposite side; and b) the size (number of vertices) of the subgraph was maximized. We refer to the vertices on the perturbation side of a densely connected subgraph as a *perturbation module*, and those on the responding side as a *response module*. The problem of finding such a subgraph from a bipartite graph belongs to the family of biclustering problems [10]–[12], which are NP-hard. There are many approximate algorithms for solving the problem (see the review by Madeira *et al.* [12]), but our formulation has distinct objectives, which allow us to specify the degree of connectivity between perturbation and responding modules. We have developed and implemented a greedy algorithm, referred to as the maximal bipartite subgraph with expected connectivity (MBSEC) algorithm, to solve this problem (see Methods).

We performed experiments to test the following two hypotheses: 1) using functional-module-based graphs as inputs for a dense-subnetwork searching algorithm would enhance the capability of identifying signaling pathways; and 2) specifically pursuing high density subgraphs enhances the capability of finding signaling pathways. To test the first hypothesis, we applied an algorithm referred to as the statistical-algorithmic method for bicluster analysis (SAMBA) by Tanay *et al.* [8] to assess the impact of different inputs on the quality of perturbation-response modules. SAMBA is a well-established algorithm that solves the biclustering problem under a bipartite graph setting, which is similar to our problem setting. We first applied the SAMBA (implemented in the Expander program, v5.2), with default settings, to the global bipartite graph consisting of all 5,289 responding genes and 300 perturbations, which returned a total of 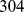 subgraphs. We then applied the SAMBA program to each of the functional-module-based graphs, and a total of 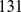 subgraphs were returned. To test the second hypothesis, we applied the MBSEC algorithms to the same functional-module-based graphs as in the previous experiment, using the following parameter settings: 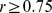 and 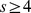. The experiment identified a total of 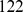 subgraphs that satisfied the requirements.

We assessed the overall quality of a perturbation (or a responding) module by determining the functional coherence score of the module using the method previously developed by our group [18]. This method measures the functional relatedness of a set of genes based on the semantic similarity of their functional annotations, and provides a p-value of the coherence score of a gene set. The key idea of this method is as follows: given a set of genes, map the genes to a weighted graph representing the ontology structure of the GO, in which the weight of an edge reflects the semantic distance between the concepts represented by a pair of GO terms; identify a Steiner tree that connects the GO terms annotating these genes and measure how closely the genes are located within the graph using the total length of the tree; apply a statistical model to assess if the genes in the set are more functionally related than those from a random gene set. A gene set with a small p-value would indicate that the functions of the genes are coherently related to each other.

**Figure 2:**
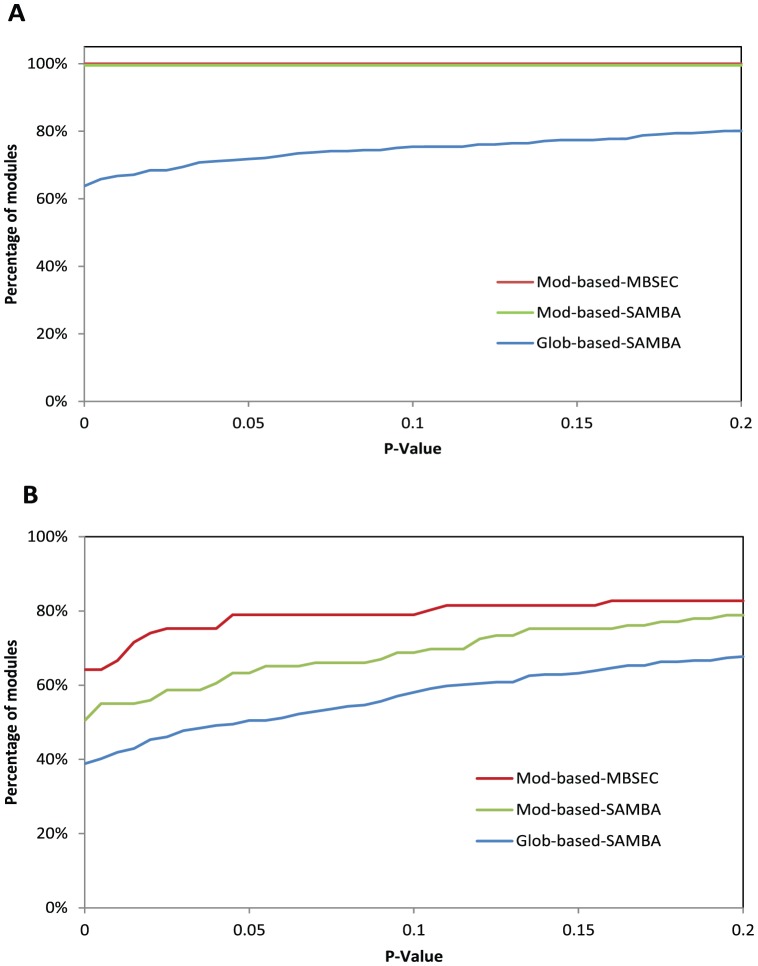
Functional coherence of modules. **A**. The cumulative distribution of functional coherence p-values of the responding modules identified by different methods: MBSEC with module-based input graphs (red); SAMBA with module-based input graphs (green); and SAMBA with the global input graph (blue). **B**. The cumulative distribution of functional coherence p-values of the perturbation modules identified by different methods: MBSEC with module-based input graphs (red); SAMBA with module-based input graphs (green); and SAMBA with the global input graph (blue).

Figure 2 shows the results of functional coherence analysis of responding modules (Panel A) and perturbation modules (Panel B) by plotting the cumulative distribution of the modules based on their p-values. Panel A shows that all responding modules returned by our MBSEC algorithm, as well as those returned by SAMBA with functional-module-based graphs as input, were assessed as functionally coherent. This is not surprising, since all the input modules were functionally coherent (p-value 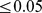), and therefore the returned responding modules, which were sets of the input modules, were likely to be coherent. In comparison, when using the global perturbation-response bipartite graph as input, about 70% of the responding modules identified by SAMBA were assessed to be coherent. The results indicate that, while the SAMBA algorithm is capable of identifying biclusters with coherent responding modules, a high percentage of returned responding modules contains a mixture of genes involved in diverse biological processes.

Since the goal is to find perturbation instances that likely constitute a signaling pathway, it is more interesting to inspect if the genes in a perturbation module are coherently related. We assessed the functional coherence of the perturbation modules returned from the three experiments for the impact of different inputs and algorithms on the results (see Panel B of Figure 2). A higher percentage of perturbation modules was found to be functionally coherent when functional-module-based graphs were used as inputs for SAMBA, as compared with those from the SAMBA with a global graph, indicating that, indeed, perturbation instances densely connected to a functionally coherent responding module were more coherent themselves; i.e., they were more likely to function together. When comparing the results from the MBSEC algorithm with those from the SAMBA, our algorithm returned the highest percentage of functionally coherent perturbation modules. The results indicate that, when inputs are the same, specifically pursuing high density subgraphs enhances the quality of identified perturbation modules.

**Figure 3:**
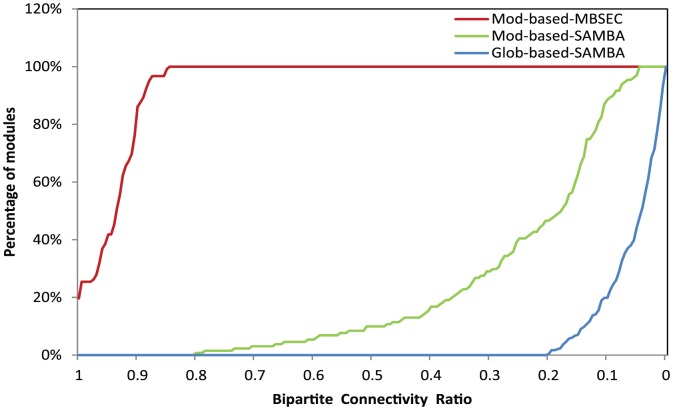
Subgraph connectivity. Cumulative distribution of within bipartite subgraph connectivity of the modules identified in three experiments: MBSEC with module-based input graphs (red); SAMBA with module-based input graphs (green); and SAMBA with global input graph (blue).

We further inspected the within-subgraph connectivity, determined as the number of edges within a subgraph over the number of maximal possible edges (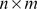, with 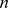 and 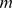 representing the number of vertices on each side, respectively), to investigate if the differences in functional coherence of the modules were related to the capabilities of the algorithms to find densely connected graphs. Figure 3 shows that there were striking differences in the connectivity of the subgraphs returned from the three experiments. The results also support the notion that an enhanced capability of finding densely connected perturbation-response bipartite graphs underlies the capability of identifying coherent modules.

**Figure 4:**
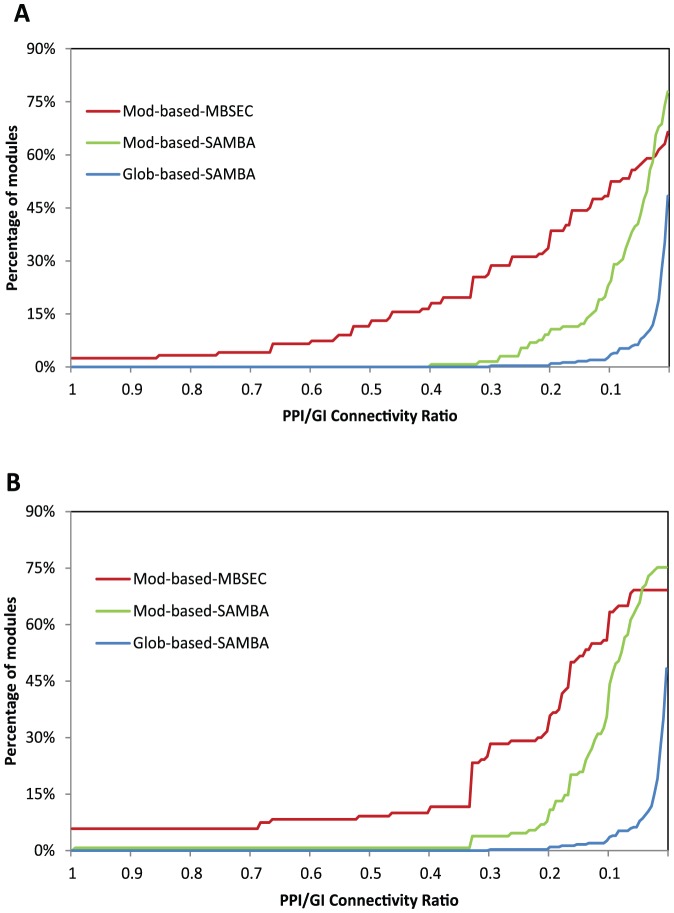
Protein-protein physical and genetic interactions within modules. **A**. The cumulative distribution of the within module PPI/GI connectivity ratios of responding modules identified by different methods: MBSEC with module-based input graphs (red); SAMBA with module-based input graphs (green); and SAMBA with the global input graph (blue). **B**. The cumulative distribution of the connectivity ratios within perturbation modules identified by different methods: MBSEC with module-based input graphs (red); SAMBA with module-based input graphs (green); and SAMBA with the global input graph (blue).

In addition to assessing the functional relationship of the genes, we further quantified and compared within module physical and genetic interactions, providing another line of evidence for assessing if genes in the modules were functionally related. Using protein-protein physical interaction and genetic interaction data from the BioGrid [19], we calculated the ratio of the number of known interactions within a module containing 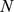 genes over the maximum number of possible interactions for the module (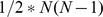). In Figure 4, we plot the cumulative distributions of modules based on their interaction ratios. The Figure shows that there are more physical and/or genetic interactions within both perturbation and responding modules identified by our methods, indicating that, indeed, the genes in these modules are more likely to function together.

Taken together, these results indicate that, by constraining the search space to *functionally coherent genes* and explicitly requiring a degree of connectivity of subgraphs, our approach enhances the capability of identifying perturbation modules in which the genes are more likely to physically interact with each other to participate in coherently related biological processes. Thus, they are likely to participate in a common signaling pathway and to carry a common signal.

### Discovering signaling pathways based on perturbation-responding subgraphs

A subgraph consisting of a perturbation and a responding module reflects the fact that the perturbation instances affected the signal controlling the expression state of the genes in the responding module. It is interesting to see if a perturbation module contains the members and/or modulators of a signaling pathway. Indeed, we found many of the identified perturbation modules corresponded to well-known signaling pathways. For example, our analysis identified a subgraph consisting of a responding module of 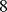 genes annotated by the GO term GO:0019236 (*response to pheromone*) and a perturbation module consisting of 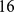 perturbation instances: {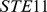, 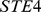, 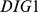, 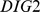, 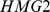, 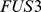, 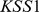, 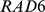, 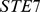, 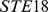, 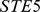, 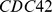, 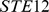, 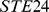, 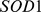, 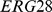}. In the list of the perturbation instances, we highlighted (with blue font) the genes that are known to be members of the well-studied yeast pheromone response pathway reviewed by Gustin *et al.* [2], which listed 20 gene products as the members of the pathway. In the study by Hughes *et al.* [4], 12 out of those 20 genes were deleted. We found that 10 out of these 12 perturbation instances were included in the perturbation module of this subgraph. This result indicates that our approach is capable of re-constituting the majority of the genes involved in the pheromone signaling pathway. Inclusion of ergosterol metabolism enzymes *ERG28 and HMG2* in the perturbation module indicates that our approach can also identify the modulators of a signaling pathway.

**Figure 5:**
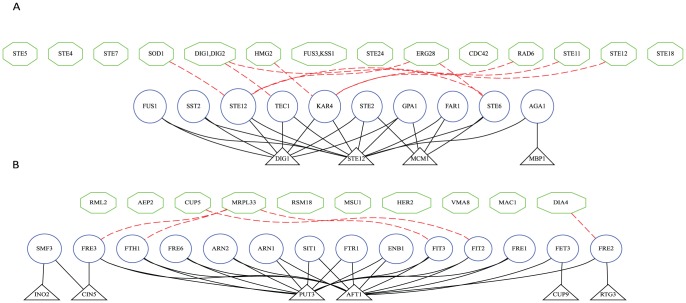
Example perturbation-responding subgraphs. Two example subgraphs are shown: **Panel A**, GO:0019236 (*response to pheromone*) and **Panel B**, GO:0006826 (*iron ion transport*). For each subgraph, the perturbation instances (green hexagons) are shown in the top tier; responding genes (blue circles) are shown in the middle tiers; and the transcription factor modules (grey triangles) are shown in the bottom tier. To avoid an overly crowded figure, a red dash line indicates that a perturbation instance and a responding gene are NOT connected.

In addition to “rediscovering” the known signaling pathways, analysis of subgraphs obtained in this study led to novel hypotheses. For example, in one subgraph, the responding module was annotated with GO:0006826 (*iron ion transport*), and consisted entirely of genes involved in cellular iron homeostasis, including iron transporters and ferric reductases (shown in Panel B of Figure 5). These genes are known to be primarily regulated by the iron-responsive transcription factor *Aft1p*, and partially comprise the iron regulon in yeast [20]. Intriguingly, the perturbed gene set consisted largely of proteins involved in mitochondrial translation, including gene products involved in mitochondrial ribosomal subunits (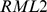, 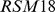, 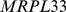), translation (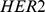, 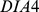, 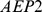), and RNA processing (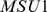). These data lead to a novel hypothesis that perturbation of mitochondrial protein synthesis will lead to changes in the iron-sensing process. In fact, such a link has only recently been suggested, in that iron-sulfur complex synthesis in mitochondria, which requires a set of 10 distinct protein components [21], directly impacts cellular iron uptake and utilization [22], [23]. Indeed, these data provide a rationale for the new hypothesis that mitochondria translation plays an essential role in cell iron homeostasis through iron-sulfur complex synthesis.

We have visualized all the perturbation-responding module pairs identified in our experiments, and show the results on the supplementary website. The data allow readers, particularly yeast biologists, to inspect the results and assess the quality of the modules, and, more importantly, to explore new hypotheses regarding yeast signaling systems. In Figure 5, we show the subgraphs related to GO:0019236 (*response to pheromone*) and GO:0006826 (*iron ion transport*). Here the perturbation instances (green hexagons) and responding modules (blue circles) are shown in two tiers. Due to the fact that the connections between the perturbation and the responding module are very dense, and thus would interfere with visualization, we conversely indicate perturbation instances and responding genes that were NOT connected, shown as the red dash-lines in the Figure. Using a set-cover-based algorithm [24], we further identified transcription factor modules (red triangles) that are likely responsible for the co-expression of the genes in the responding modules. Including TF information in data visualization further enhances the interpretation of the subgraphs. For example, the fact that each responding module in this Figure is connected (thus potentially regulated) by a TF module further strengthens the hypothesis that the genes are co-regulated as a unit responding to a common signal.

### Revealing the organization of cellular signals

**Figure 6:**
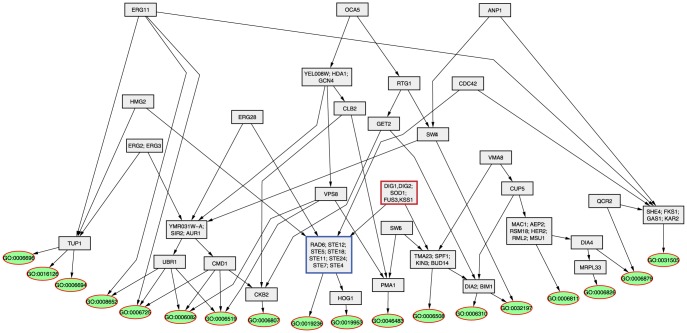
Organizing perturbation instances and responding modules. In this graph, responding modules are represented as green oval nodes, with each being annotated by a GO term. The rectangle nodes are perturbation nodes, which may contain one or more genes that share a common set of responding modules.

Our approach enabled us to use responding modules to reflect major signals in a cellular system and the perturbation instances that affect these signals. We have found that many perturbation instances were involved in multiple perturbation-response subgraphs, indicating that the signal affected by such perturbation instances was connected to multiple signals through cross-talks. This observation offered us an opportunity to further investigate the organization of cellular signals by studying what signals each perturbation instance affects, and how the signals are related to each other. For example, it is interesting to investigate whether a set of perturbation instances affects a common set of responding modules—that is, that the information encoded by these genes is identical—so that we can group them as a signaling unit. Similarly, it is of interest to investigate whether the responding modules (signals) affected by one perturbed gene are a subset of those affected by another perturbed gene, and to utilize such a relationship to organize the signals. The latter task is closely related to that addressed by the nested-effect model [6], which aims to capture the hierarchical relationship among perturbation instances based on the genes they affect. Since the nested effect model used an individual gene as a responding unit, the scale of the problem became intractable (exponential), and a Markov chain Monte Carlo algorithm was employed. In contrast, our approach used conceptualized responding modules, providing two advantages: 1) the projection of high-dimensional data at the gene level to a low-dimensional and semantic-rich concept level reduces the complexity of the task; and 2) the unique annotation associated with each module renders the task of determining subset relationships among perturbation instances a trivial task. These characteristics enabled us to develop a polynomial algorithm (see Methods) to organize the perturbation instances into a DAG. In such a graph, each node is comprised of a set of perturbation instances that share common responding modules, i.e., a signaling unit; an edge between a pair of nodes indicates that the signals affected by the parent node subsume those carried by the child node. We collected all perturbation modules that contained at least 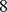 perturbation instances, and these organized perturbation instances into a DAG, as shown in Figure 6.

Inspecting the perturbation nodes that included multiple genes, we found that the genes in these nodes tend to participate in coherently related biological processes, and that they often physically interact with each other at high frequencies (data not shown). For example, one perturbation node (highlighted with a blue border in Figure 6) contained multiple sterility (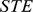) genes, a set of well-studied genes that mediates pheromone signaling in yeast, and they shared common responding modules annotated with the functions “response to pheromone” (GO:0019236) and “sexual reproduction” (GO:0019953). Thus, our method is capable of identifying perturbed instances whose information can be encoded using a one-bit signal—a switch affecting expression of the genes responding to pheromone signaling.

Visualization of the relationship of perturbation instances in a DAG enables a biologist to investigate how signals are combined to generate a cellular response. For example, there is a perturbation node (highlighted with a red border) in Figure 6 containing 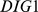, 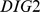, 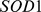, 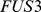, and 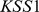, all of which, except 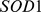, are involved in MAPK activities. Our results show that there is a path connecting this node to the aforementioned 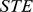 node, and then further to the “respond to pheromone” responding module, indicating that the gene products of the two nodes work together to transmit signals in response to pheromone. Indeed, it is well known that MAPK activities are required in the pheromone signaling pathway [2], [3]. Yet, our results reveal that the MAPK node, besides carrying information about pheromone response, also affects the biological processes of “proteolysis” (GO:0006508).

Another interesting observation is that the hierarchical organization of the perturbation instances reflects their relative position in a signaling cascade. For example, perturbations of ergosterol metabolism genes *ERG2, ERG3, HMG2, ERG11*, and *ERG28* tend to have a broad impact on different signals, including the pheromone response pathway. This is understandable: as a critical component of the plasma membrane, ergosterol influences the organizational compartments of the plasma membrane, such as lipid rafts [25], which in turn affect the organization of signaling molecules in the membrane. As such, perturbation of these genes has a broad impact on diverse cellular signals. Our results indicate that *HMG2* and 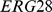 are connected to the 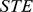 node to influence the expression of the pheromone-responding module. The role of ergosterol metabolism in modulating pheromone response signaling has only recently been studied by Jin *et al.* [26]. More interestingly, our results indicate that perturbation of distinct enzymes of ergosterol metabolism leads to distinct cellular signals, presumably by perturbing the production of distinct species of ergosterols. The view that distinct lipid species encode/regulate disparate signals is widely accepted in the lipidomics research domain [27].

## Summary

In this study, we developed a proof of concept framework for unifying knowledge mining and data mining to conceptualize the findings from systematic perturbation experiments in order to enhance the capability of identifying signal transduction pathways. The innovations of our approach are reflected in the following aspects: 1) an ontology-driven approach for identifying functional modules from a genes list in a dynamic and data-driven (instance-based) manner, and projecting molecular findings to a conceptual level; 2) innovative formulation of the biclustering problem in terms of a constrained search space and new objective functions; and 3) a novel graph algorithm that enables organizing signaling molecules at a system level in a tractable manner for the first time. We have demonstrated that conceptualization of cellular responses to systematic perturbations enhances the capability of identifying perturbation instances that participate in specific signal transduction pathways. To the best of our knowledge, this is the first report of a computational framework capable of automatically assimilating the information from systematic perturbation data to reveal the architecture of a cellular signaling system at a *conceptual* level, so that it can be readily interpreted by biologists to gain insights into a system.

More importantly, conceptualization of experimental results is a critical step towards the ultimate goal of systems biology—acquiring computable knowledge from experimental data for reasoning and hypothesis generation. Our results already laid the foundation for deriving abstract knowledge. For example, one can translate a path from a perturbation node to a responding module in Figure 6 into a rule, as follows: “*if genes involved in MAPK signaling are perturbed, genes involved in pheromone responses will be differentially expressed.*” A rule like this represents the relationships between perturbed genes and responding genes at a conceptual level. Equipped with rules and facts, a computing agent can then make the prediction that perturbation of a newly discovered gene may lead to the differential expression of genes involved in *pheromone responses*, if the gene is found to be involved in *MAPK signaling*. Ongoing research is devoted to acquiring and representing facts, assertions, and rules from systems biology data in an accurate and generalizable manner.

## Materials and Methods

The microarray data from the systematic perturbation experiments by Hughes *et al.* [4] were collected, and differentially expressed genes responding to each perturbation were identified based on the analysis of the original paper. Given a list of differentially expressed genes responding to a perturbation instance, we represent the genes and their annotations using a data structure referred to as GOGene graph [28]. In such a graph, a node represents a GO term and a directed edge between a pair of nodes reflects an “is-a” relationship between the GO terms; in addition, each node keeps track of the genes it annotates. Therefore, the graph contains information on both GO terms and genes. The procedure for searching for summarizing GO terms iterates through the following steps: 1) perform an enrichment analysis [29] for each leaf GO term among the instance-specific responding genes; 2) select the GO term with the biggest p-value (least enriched) and merge its genes to the parent node with the shortest semantic distance as defined by Jin *et al.* [30]; 3) trim the term from the graph; 4) repeat the above procedures. We stop trimming a GO term once it is significantly enriched (p-value
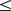
0.05) and the genes summarized by the term remain functionally coherent [18]. Its associated genes are treated as a functionally coherent module; otherwise, all non-significant terms would eventually be merged to the root node of the GO hierarchy and their associated genes are deemed as not coherently related.

To assess the functional coherence, we applied the method developed by Richards *et al.* [18]. In this approach, the ontology structure of the GO is represented as a weighted graph, in which an edge weight represents the semantic distances between a pair of GO terms. Given a list of genes, we associate them to their GO annotations, and identify a Steiner tree that connects them all. Using the total length of the Steiner tree as a score reflecting the functional relatedness of the genes, we apply a statistical model to assess the probability of observing such a score if sets with the same size are randomly drawn from the yeast genome. See paper [18] for details.

**Figure 7:**
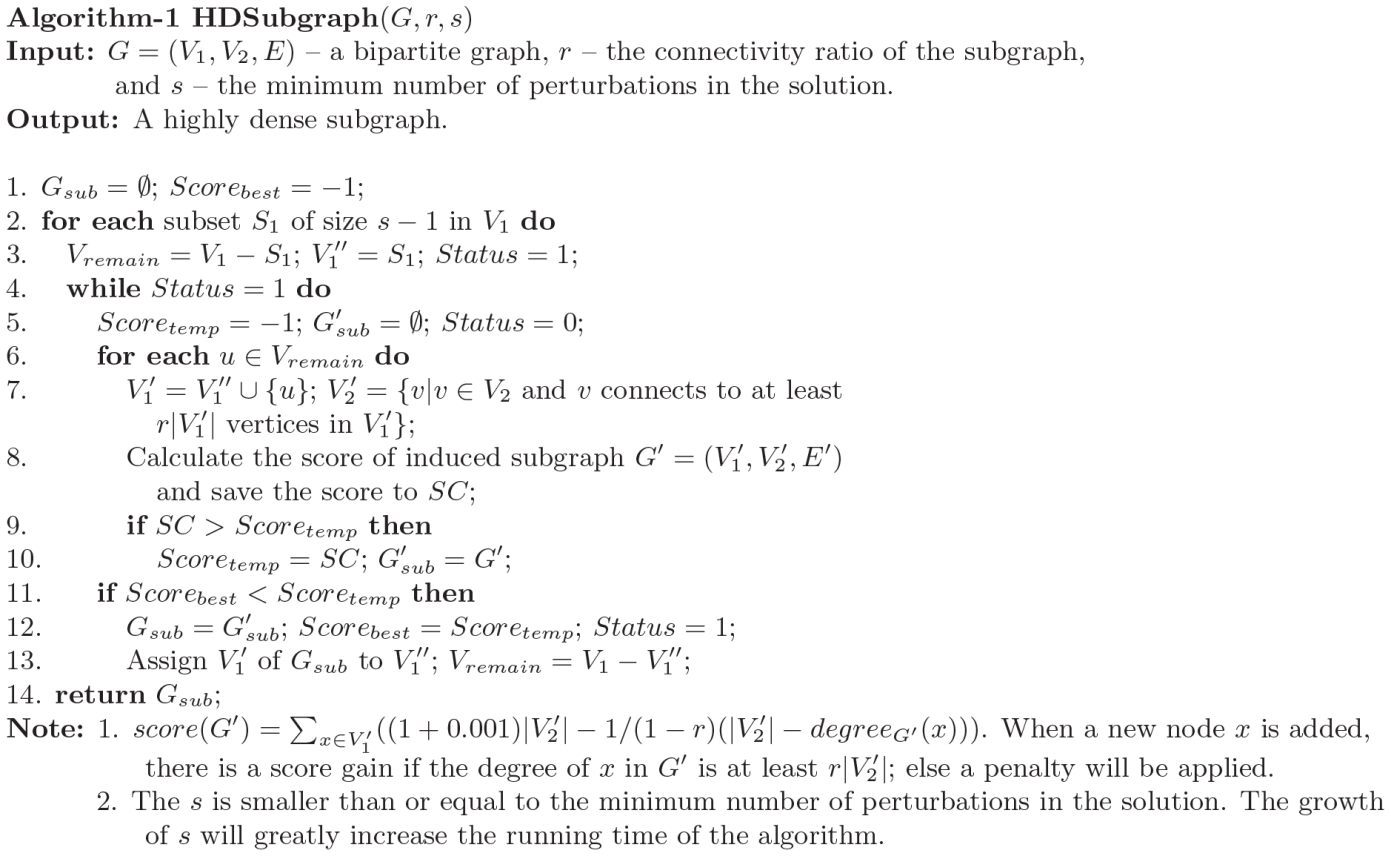
Greedy algorithm to find the highly dense bipartite subgraph.

To search for a densely connected perturbation-responding subgraph in a bipartite graph, we formulated the task as follows: given a bipartite graph 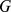, find a subgraph 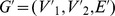 of 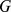 that satisfies the following conditions: 1) 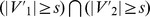, where 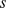 is a user defined threshold for cluster size; 2) each vertex in 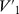 connects to at least 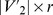 vertices in 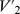, and each vertex in 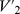 connects to at least 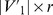 vertices in 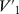, where the parameter 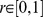 is a connectivity ratio defined by users; and 3) the size of the subgraph (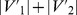) is maximized. We set the parameters as follows: 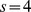 and 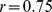. The algorithm for searching for the subgraph is shown in Figure 7.

**Figure 8:**
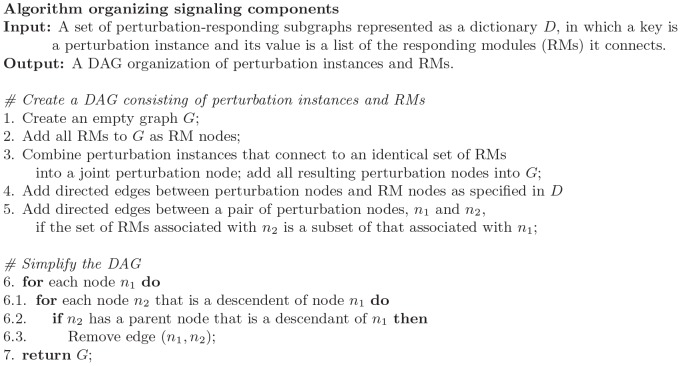
Algorithm for organizing perturbation instances and RMs.

To organize perturbation instances based on their signals, we developed an algorithm to organize the perturbed instances into a DAG. In such a graph, there are two types of nodes: responding module nodes and perturbation nodes. Our algorithm groups perturbation instances that share identical responding modules into a common perturbation node, a signaling unit, and connect the perturbation node to its corresponding responding modules. The algorithm further organizes perturbation nodes such that, if signals by a perturbation node subsume those of another, a directed edge pointing to the subsumed node is added between them. The algorithm is shown in Figure 8.

### Supplementary Website

http://pubreview.dbmi.pitt.edu/Supplement/functional_modules.html
